# Basing Turkey Lighting Programs on Broiler Research: A Good Idea? A Comparison of 18 Daylength Effects on Broiler and Turkey Welfare

**DOI:** 10.3390/ani6050027

**Published:** 2016-04-25

**Authors:** Karen Schwean-Lardner, Catherine Vermette, Marina Leis, Henry L. Classen

**Affiliations:** 1 Department of Animal and Poultry Science, University of Saskatchewan, Saskatoon, SK S7N 5A8, Canada; cjv425@mail.usask.ca (C.V.); hank.classen@usask.ca (H.L.C.); 2 Western College of Veterinary Medicine, University of Saskatchewan, Saskatoon, SK S7N 5B4, Canada; marina_leis@hotmail.com

**Keywords:** welfare, behaviour, melatonin, circadian rhythms, mobility

## Abstract

**Simple Summary:**

Altering daylength in a poultry management program is a simple tool that can have immense impacts on productivity and bird welfare. It is not uncommon for lighting data derived from broiler research to be extrapolated to turkey production. This review of two studies (one with broilers and the second with turkeys), completed in the same research facility using the same lighting programs, shows evidence that some, but not all responses to graded daylengths are similar between these two species. It defines that daylength choices for turkeys should be based on research conducted with turkeys.

**Abstract:**

Daylength used as a management tool has powerful implications on the welfare of both broilers and turkeys. Near-constant light results in many detrimental impacts, including lack of behavioural rhythms and circadian melatonin rhythms. Both are suggestive that sleep fragmentation could result in birds reared on long photoperiods, which can lead to the same negative health and physiological responses as total sleep deprivation. An indirect comparison of the welfare implications of graded levels of daylength on broilers and turkeys clearly indicate that long daylengths depress welfare by increasing mortality, reducing mobility, increasing ocular pathologies and changing behaviour in both species. Furthermore, long daylengths change melatonin secretion patterns and eliminate behavioural and melatonin circadian rhythms, which were measured in broilers in these works. However, feather pecking in turkeys was reduced when birds were exposed to long daylengths. Exactly how much darkness should be included in a management program to maximize welfare will depend on the species, the age of marketing, and in turkeys, bird gender.

## 1. Introduction

Lighting programs, including the length of the photoperiod, have immense potential to impact the welfare and productivity of poultry. Vision may be the most important sense that birds have [1] and all aspects of light (duration and pattern, intensity and wavelength) have the potential to impact behaviour and physiology of the bird [2]. Light itself is a powerful zeitgeber—it is one of the primary factors that drives circadian rhythms in many species [3,4,5,6]. This has been recognized in some areas of the world. Europe requires birds be given a light/dark period that results in circadian rhythms for the majority of their life (EC Council Directive 2007/43/EC). However, many parts of the world have no specific requirement for the inclusion of dark in a broiler or turkey photoperiod.

Melatonin is one such neurohormone produced in a circadian fashion [7]. Since light can repress melatonin production, the peak production should occur during the scotophase, and the trough of the wave during the photophase [8]. Melatonin has a number of functions, including regulating other circadian rhythms and production of other hormones in the body, including those related to growth and immune function, as well as modulating sleep [7,8]. 

Sleep deprivation in birds can have negative impacts on the welfare of poultry [9]. In many species, it is thought to alter brain function and reduce alertness [10], increase pathological disease [11], and increase stress and reduce immune function [12] to list some of the effects. Sleep fragmentation, which occurs with repeated awakenings, has been shown to result in similar responses to total sleep deprivation [13,14]. Hence, behavioural rhythms for birds are also important, and many poultry rhythms have been shown to function in a circadian fashion, including sleeping [15] and feeding [16]. Lack of such rhythms could result in sleep fragmentation.

The responses of birds to daylength have been previously documented, with a plethora of studies conducted in the 1970s–1990s. The majority of research at that time was conducted on broiler responses to daylength, with the focus primarily to examine production responses. Research into the effect of daylength on turkeys was less common, primarily because the work is expensive, flock turnover is less frequent, and the studies are simply more difficult because of the bird sizes. As a result, it is not uncommon for management decisions for turkeys to be made on data gathered from broiler experiments.

While broilers and turkeys are both avian species used for meat production, there are marked differences which could impact how the birds respond to varying hours of daylength/darkness per day. These could result from evolutionary differences or potential spectral sensitivity differences between the species, but an obvious difference is likely the impact of age—while broilers tend to be slaughtered for meat production in North America between 30 and 64 days of age, turkey grow-out periods are extending up to 25 weeks of age. Therefore, the latter species may respond to light and dark in ways broilers do not have the opportunity to.

It is very difficult to compare published daylength research studies within species because of the variability that occurs in the materials and methods from study to study, including factors such as different barn environments, different light sources, different management styles, *etc.* It is even more difficult to compare the responses of various strains of poultry to daylength across scientific studies. The authors of this paper have conducted two experiments that allow an indirect comparison of the impacts of daylength on broilers and turkeys, while still recognizing that many factors must vary simply because of the nature of the different birds. In the first experiment (repeated four times (four trials)), broilers were exposed to either 14 h of daylength and 10 h of dark (14 L:10 D), 17 L:7 D, 20 L:4 D or 23 L:1 D up to 49 d of age, with two replications of independently environmentally controlled rooms in each of the four trials. Following that work, a similar study (repeated twice) was conducted using the same research facilities, with turkeys to 18 weeks of age, again exposed to the same lighting programs. Each of these studies have previously been published as individual works [17,18,19,20,21,22,23], but this review will compare the effects on the two species.

Detailed materials and methods for these studies are outlined in the corresponding journal articles [17,18,19,20,21,22,23]. To briefly outline these—the floor-barn facility used for both the broiler and turkey experiments is composed of eight individual rooms (two room replications per trial, with four trials for broiler studies and two trials for turkey studies), each with independent environmental control systems. The lighting was supplied by incandescent bulbs, with intensity ranging between 40 and 10 lux for broilers, and 40 and 2 lux for turkeys, depending on age. All treatments within species were exposed to identical intensity patterns. Each room was subdivided into either 12 (2.3 m × 2 m (broiler)) or six (4.6 m × 2 m (turkey)) pens for replications of genders, and strains (broiler work only). Final estimated housing densities were 32 or 35 kg/m^2^ for broilers and turkeys respectively. Infrared cameras were mounted over one male pen in each room for video-taping bird behaviour on a 24-h basis, then videos were scan sampled at 15 min intervals for both species. For both experiments, pen weights of all birds were taken at various dates to establish growth rates and curves, and feed efficiency values. All mortality and culled birds were necropsied by the same independent laboratory (Prairie Diagnostic Services, Western College of Veterinary Medicine, Saskatoon, SK, Canada) for cause of death or morbidity. The daylengths used were 14 L:10 D, 17L:7 D, 20 L:4 D and 23 L:1 D, initiated at either 7 d (broilers) or 10 d (turkeys of age), with the dark period given in one section. Prior to program initiation, all birds were exposed to 23 L:1 D.

Broilers: A total of 16,128 broilers (male and female, Ross 308 or Ross 708) were exposed to one of the four lighting programs, over four separate trials. Both strains tested reacted similarly to daylength, and strain differences will not be discussed in this review. Gait scoring to assess mobility followed the scoring system of Garner *et al.* [24], where “0” represents a normal walking bird, and “5” a bird that cannot stand. Foot pad lesion scoring [25] scored a healthy footpad as a “0” and one with a severe lesion as a “2”. 

Turkeys: A total of 840 male and 720 female Nicholas heavy strain (85 × 700) poults (over two trials) were exposed to one of the four lighting programs. Gait scoring to assess mobility followed the scoring system of Garner *et al.* [24], where “0” represents a normal walking bird, and “5” a bird that cannot stand. Foot pad lesion scoring 2 scored a healthy footpad as a “0” and one with a severe lesion as a “2” [26].

Data for both trials were analyzed initially using a General Linear Model (Proc Mixed), with lighting nested within rooms, to test the main effects of light, gender and strain for broilers, and for light and gender in turkeys, and the their interactions. Regression analysis (Proc RSReg and Reg) was also used to better determine the relationship between graded daylength levels and each dependant variable.

## 2. Results and Discussion

**Figure 1:**
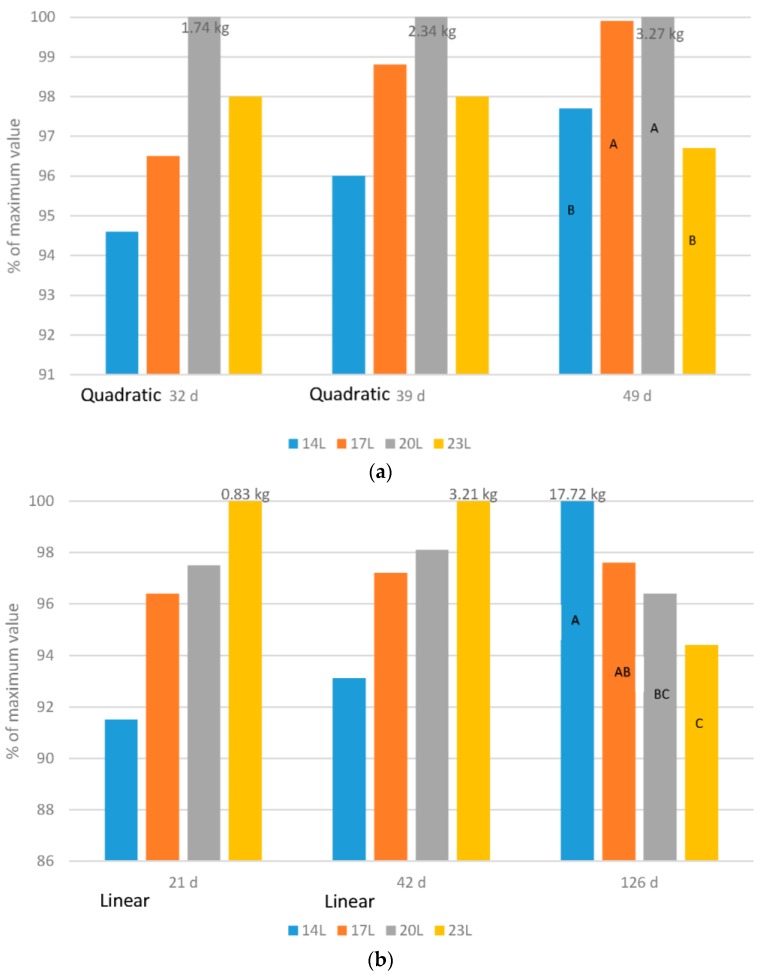
Impact of daylength on body weight. (**a**) Comparison of broiler body weight under four daylength photoperiods at three ages, with data expressed as a percentage of the maximum value within each age; (**b**) Comparison of turkey body weight under four daylength photoperiods at three ages, with data expressed as a percentage of the maximum value within each age.

**Figure 2:**
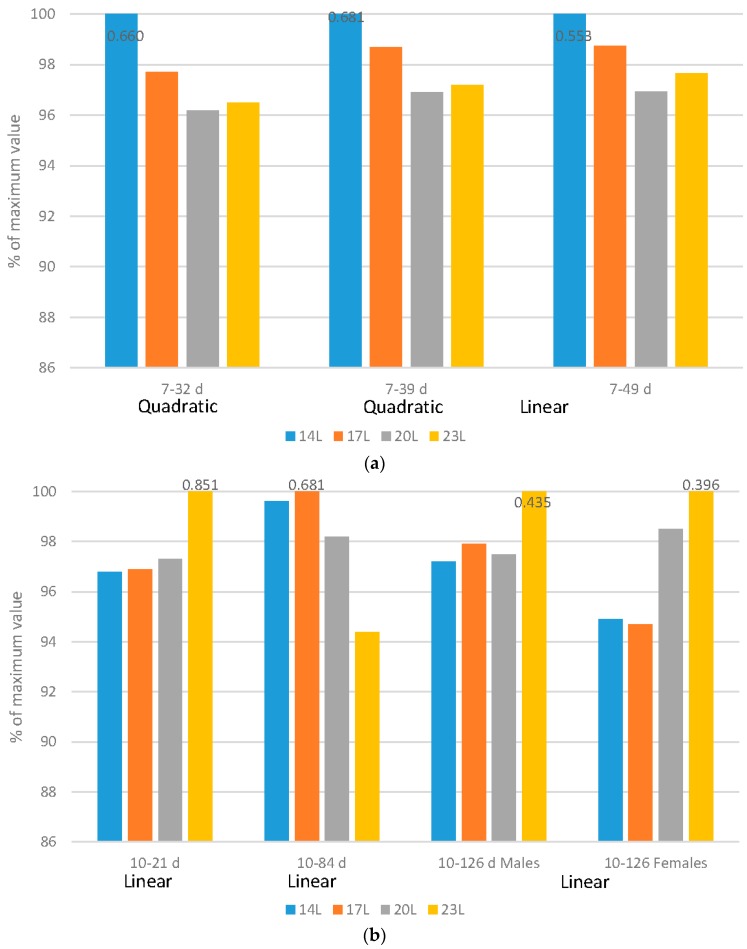
Effect of daylength on feed efficiency. (**a**) Comparison of broiler gain to feed efficiency ration (mortality corrected) under four daylength photoperiods at three age ranges, with data expressed as a percentage of the maximum value within each age; (**b**) Comparison of turkey gain to feed efficiency ration (mortality corrected) under four daylength photoperiods at three age ranges, with data expressed as a percentage of the maximum value within each age.

**Figure 3:**
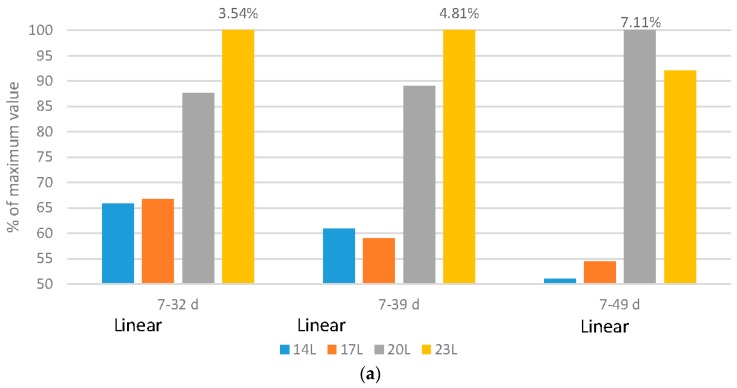

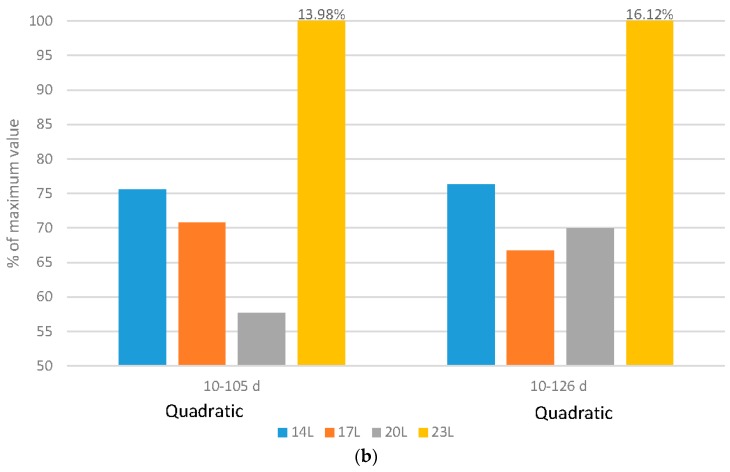
Impact of daylength on mortality and cull levels. (**a**) Comparison of broiler mortality and cull levels (% of placed) under four daylength photoperiods at three age ranges, with data expressed as a percentage of the maximum value within each age; (**b**) Comparison of turkey mortality and cull levels (% of placed) under four daylength photoperiods at two age ranges, with data expressed as a percentage of the maximum value within each age.

**Figure 4:**
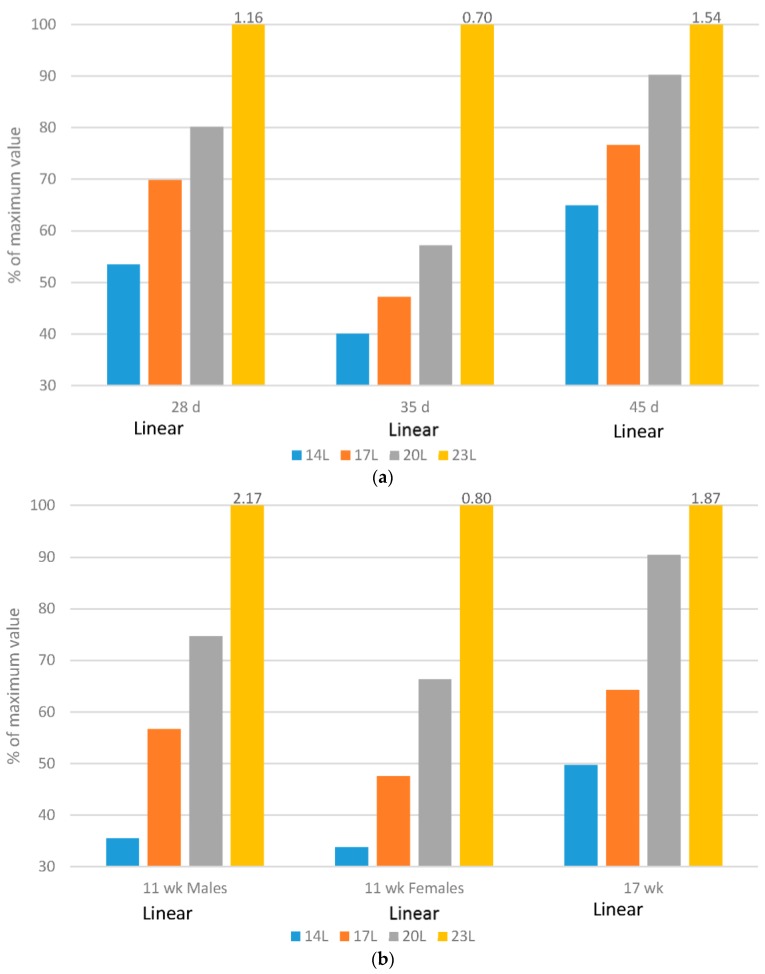
Daylength effects on mobility (measured by gait score). (**a**) Comparison of broiler mobility based on average gait score under four daylength photoperiods at three age ranges, with data expressed as a percentage of the maximum value within each age; (**b**) Comparison of turkey mobility based on average gait score under four daylength photoperiods at three age ranges, with data expressed as a percentage of the maximum value within each age.

**Figure 5:**
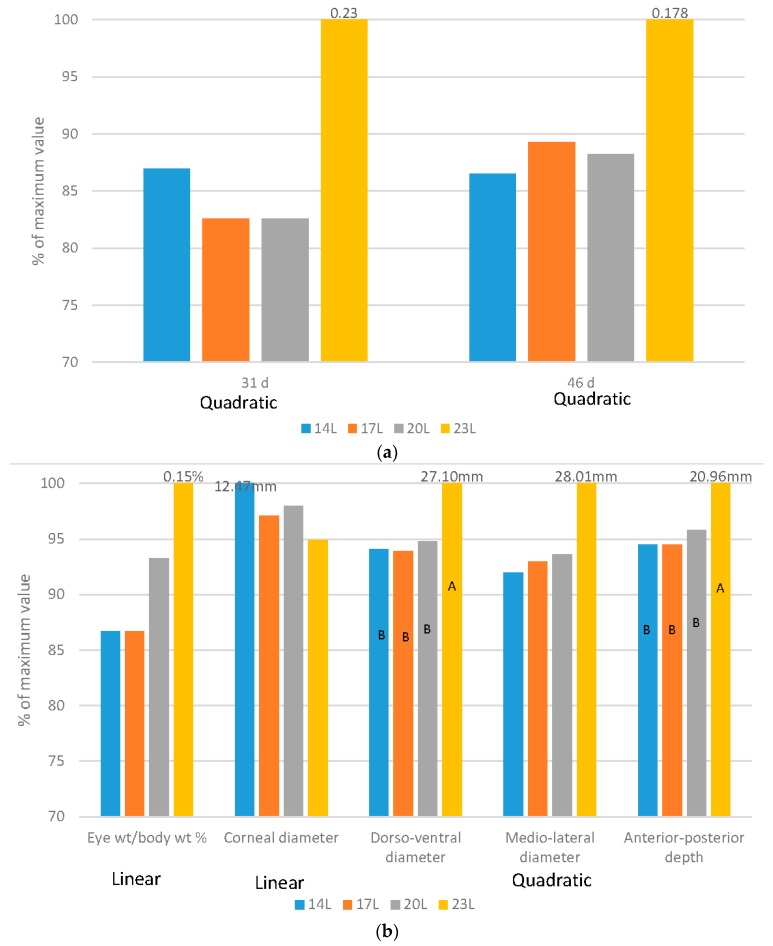
Effect of daylength on ocular measures. (**a**) Comparison of broiler relative eye weight (g/kg body weight) at 31 or 46 d of age under four daylength photoperiods, with data expressed as a percentage of the maximum value within each age; (**b**) Comparison of turkey ocular dynamics at 12 weeks of age under four daylength photoperiods, with data expressed as a percentage of the maximum value within each age.

Figure 1, Figure 2, Figure 3, Figure 4 and Figure 5 have expressed all data on a percentage of the maximum value reached in each case (with the maximum value expressed as 100%). This allows a simple comparison between the species. The actual data value is listed above each 100% bar in each figure. The labels of “Linear” or “Quadratic” define the significant statistical shape of the curve (*p* < 0.05) where applicable, with no label signifying no regression relationship.

### 2.1. Growth Rate

Poultry prefer to feed during the photophase rather than during the scotophase. By providing this visual access to feeders and waterers for long periods of time (23 L or 24 L per day), some sectors of the poultry industry believe that birds can interact in their environment when they choose, leading to increased feeding time and higher growth rates. This is one reason why the use of such long daylengths is not uncommon for poultry production in some sectors of the world. 

Broilers: Adding darkness to a broiler photoperiod program altered the growth curve of birds, so that in early life, growth was slower (Figure 1a). However, at older ages (32, 39 or 49 d) body weight of birds reared under 23 L was never the heaviest. If indeed the birds could examine their environment as some think occurs in long daylength photoperiods, then this would not be expected. Furthermore, as the birds aged, compensatory gain occurred so that by 49 d of age, birds given either 4, 7 or 10 h of darkness were heavier than those given 23 L. In these works, birds exposed to 7 h of darkness weighed as much as those given only 4 h.

Turkeys: The effect of daylength on turkey growth rate was also age dependant (Figure 1b), hence adding darkness shifted the growth curve as in broilers. Body weight at young ages (21 and 42 d of age in our work) responded in a linear fashion, with the heaviest weights found in turkeys exposed to long daylengths. As the birds aged, compensatory gain occurred in turkeys given a dark period, and although an interaction existed (male response was larger than the female response), birds reared under 14 L were the heaviest and 23 L the lightest.

Adding darkness to a photoperiod shifts the growth curve for both broilers and turkeys. This allows the development of skeletal and metabolic systems prior to the requirement to support heavy weights that occur later in life, and can be a factor in the health and mortality levels of birds later in life [17,20,21]. This unexpected reduction in market body weights may also be an indicator of poor welfare. While superior productivity, such as maximum growth rate, should never be used alone as an indicator of bird well-being, poor productivity in an appropriate environment may be indicative of a negative issue.

### 2.2. Feed Efficiency

Broilers: Regardless of the flock age examined, adding darkness to a photoperiod program improved feed efficiency in either a quadratic (7–32 d or 7–39 d) or linear (7–49 d) fashion (Figure 2a). It is important to remember that as birds increase in size, feed efficiency reduces, so even though birds reared under 20 L and 23 L have similar feed efficiency ratios, correcting for the different body sizes show that the poorest efficiency occurs in birds reared under 23 L.

Turkeys: Feed efficiency, corrected for mortality changed during the grow-out period, and gender impacts were noted over the grow-out period (Figure 2b). Over very young ages (10–21 d), poults exposed to long days (23 L) had improved feed conversion ratio (FCR) (linear impact). As birds age and adjust to dark periods (10–84 d), darkness improved feed efficiency in a linear fashion. Over the 10–105 d and 10–126 d periods, genders reacted differently. Males responded with improved FCR under longer days over the 10–105 d period, but females actually had an improved feed efficiency with the use of 23 L over the 10–126 d period.

Like growth rate, the ratio that birds convert feed into growth is considered to be primarily a production trait. But, health and other factors, including activity levels of the birds [27], sexual maturity [28] can also alter feed efficiency ratios. The response of turkeys varies from that of broilers with regards to feed efficiency. This may be partially related to differences in body weight, as the feed efficiency typically declines as body size increases. In this work, the heaviest birds at 126 d were reared under 14 L:10 D. In broilers, improved feed efficiency under shorter daylengths could be due to a number of physiological changes that take place during the dark period. Metabolic rate declines which reduces energy requirements [29,30,31] activity reduces [18,20,21] and melatonin, which aids in sleep regulation [32,33] and improves feed efficiency, peaks during the dark period [20,34] This may possibly impact feed efficiency because of the impact of proper melatonin circulation on endocrine status in the body [35]. A primary difference between the two species is age, and turkeys are closer to the age of sexual maturity than are broilers. In this work, mean testicular weight was measured in the turkeys (% of body weight) as a proxy for sexual maturity, and turkeys exposed to 23 L had a higher percentage (0.17%) as did those exposed to 14 L (0.09%). This would support the suggestion that sexual maturation plays an important part in overall feed efficiency for turkeys.

### 2.3. Mortality and Cull Levels

Broilers: The health of broilers, measured by the percentage of mortality and culled birds, improved linearly with the addition of darkness (Figure 3a). The fast growth of commercial broilers is often suggested to cause a reduction in the health of broilers, but this data suggest that other factors may also be important. By using darkness to shift the growth curve, birds under 14 L and 17 L, although as heavy as those reared under 23 L, had significantly lower mortality and morbidity rates [19]. Adding darkness linearly reduced metabolic and skeletal disorders (metabolic—1.23%, 1.20%, 1.82% and 1.73%, and skeletal 0.368%, 0.25%, 0.50%, and 0.70% of placed for 14L, 17L, 20L and 23L birds respectively over the 7–32 d period; metabolic—1.42%, 1.35%, 2.00% and 2.36% and skeletal—0.77%, 0.40%, 0.86% and 1.11% for 14 L, 17 L, 20 L and 23 L birds respectively over the 7–39 d period), again suggesting that shifting away from early weight deposition is beneficial to bird health. The effects were more pronounced over the 7–49 d period for the 14 L, 17 L, 20 L and 23 L birds, with a linear reduction in metabolic disorders (1.91%, 1.54%, 2.96% and 2.99% of placed respectively) and skeletal disorders (0.36%, 0.73%, 1.71% and 1.48% of placed respectively) and quadratic responses in infectious disorders (0.65%, 1.44%, 2.01% and 1.30% of placed respectively).

Turkeys: The level of mortality and culls under the various daylength programs responded in a quadratic fashion (Figure 3b). The highest level was noted under 23 L over both the 10–105 d and 10–126 d periods. Adding darkness to the photoperiod reduced the levels of skeletal disorders in a linear fashion (4.44%, 2.92%, 5.14% and 6.62% of placed for 14 L, 17 L, 20 L and 23 L respectively), but increased the level of injury or death to injurious pecking (2.37%, 1.60%, 1.18% or 0.83% of placed for 14 L. 17 L, 20 L or 23 L respectively).

Undoubtedly, mortality and morbidity are strong indicators of bird welfare. These studies have shown that darkness reduces these variables regardless of the species. The difference may arise with regard to the amount of darkness. While broiler mortality responds with a linear reduction in these variable levels, turkeys do not, and 10 hours of darkness may not reduce mortality/morbidity beyond that which can be accomplished with 4 or 7 hours. The reasons for the improved health in both species may be many, including improved health due to exercise, alterations to the growth curve, improved immune function [36], stronger circadian rhythms [20], and the beneficial impact of proper sleep [5,20].

### 2.4. Mobility 

Broilers: In this work, average gait score increased, indicating poorer mobility, with increasing daylength (Figure 4a). Variability of the measures, measured by pooled standard errors of the means, was 0.041, 0.023 and 0.047 for broilers scored at 28, 35 and 45 d respectively.

Turkeys: The impact of daylength on mobility was similar in turkeys as compared to broilers. At 11 weeks of age, an interaction existed between daylength and gender with respect to average gait score, with the males experiencing a larger negative impact of long daylengths than females (Figure 4b), which might be attributed to heavier tom weights. At older ages, the response was similar for both genders, but once again, longer daylengths resulted in poorer mobility. Variability, measured again by pooled standard errors of the means, was 0.075 and 0.077 for turkeys scored at 11 and 17 weeks respectively, indicating more variability in the measure for these birds as compared to broilers. 

Regardless of the species, mobility improves linearly with added darkness, with the largest improvement in this work occurring when birds (broilers or turkeys) were exposed to 10 h of darkness, per day. Previous research with broilers [37] and turkeys [38,39] has shown that birds with moderate to severe lameness experience pain, and negative affective states undoubtedly reduce welfare. Lameness also changes behavioural patterns, as inactivity increases and exercise decreases [40], likely related to pain.

### 2.5. Carcass Defects

Broilers: A positive relationship between increasing daylength and severity of footpad lesion scores was found at both 28 d (0.28, 0.36, 0.43 or 0.48 for 14 L, 17 L, 20 L and 23 L respectively) or 35 d (0.48, 0.50, 0.60 or 0.56 for 14 L, 17 L, 20 L and 23 L respectively) of age. It is interesting however, that there was no effect of daylength when birds were scored again at 45 d of age.

Turkeys: Daylength did not significantly alter average turkey foot pad lesion score at 11 weeks (1.09, 1.50, 0.89 and 1.11 for 14, 17, 20 and 23 L respectively) or 17 weeks (1.07, 1.50, 1.33 and 1.33 for 14, 17, 20 and 23 L respectively). At 17 weeks, a linear decline in the percentage of birds falling in Score 1 (minor in nature) was noted with increasing daylength, and although not significant, a numeric increase was noted under the more severe scores with increasing daylength. A significant, linear relationship was noted between daylength and the presence of breast buttons at 11 weeks of age, with the highest percentage of birds reared under 23 L (2.50%, 5.00%, 8.33% and 15.83% of birds for 14, 17, 20 and 23 L respectively).

Footpad dermatitis in poultry is influenced by many factors, including litter moisture, diet, bird density, skin thickness, bird type, *etc.* [41,42,43,44,45]. Daylength does have an impact, but likely not as strong as that of litter moisture for example. We can hypothesize a number of reasons to explain why darkness may impact skin lesions. Broilers and turkeys given a dark period in their photoperiod program are more active [18,21], and this could result in more turnover of litter, hence allow more drying to take place. Since the birds are more active over a 24 h period when given a dark period, this could also mean less contact with litter, and combined with more movement of litter, could explain this. Furthermore, diurnal variation in skin thickness has been demonstrated in some species, including humans [46] and rats [47], and the latter authors related skin thickness to melatonin production. In this work, broilers exposed to 23 L demonstrated a reduction in average melatonin production (Table 1) and a weaker melatonin rhythm over a 24 h day [20] than broilers given a dark period, and hence this may relate to lesion scores. 

### 2.6. Ocular Health

Broilers: Regardless of the age measured, increasing daylength resulted in a quadratic response in the relative weight of eyes to body weight (g/kg body weight) (Figure 5a). It is interesting that the data are similar when birds were given either 4, 7 or 10 h of darkness, and that the 1 h of darkness alone resulted in an increased ratio.

Turkeys: Altering daylength significantly impacted the size and shape of the turkey eye (Figure 5b). Birds reared on longer daylength at 12 or 18 weeks of age had a linear increase in the percentage of eye weight as compared to body weight. The eye shape itself changed dramatically in turkeys. The diameter of the cornea decreased, and the dorso-ventral diameter, medio-lateral diameter and anterior-posterior depth was increased (Figure 5) [22]. In addition, these same birds reared under 23 L, as compared to 14 L, had evidence of ocular pathology, including reduced number of nuclei in both the outer and inner nuclear layer, a reduction in the thickness of the choroid as well as a significant increase in the incidence of cataract [23].

Eye growth is related to circadian rhythms, and it is clear from both data sets that at least 4 h of darkness is required to develop this rhythm in both broilers and turkeys. While we cannot say that welfare was compromised in the broilers reared under 23 L with respect to eye health (as only eye weight was monitored), we can hypothesize tthat this degree of ocular pathology may be a welfare issue. The added length of time that turkeys live in their environment demonstrate significant welfare challenges with regards to eye health, particularly with the development of cataracts, which can lead to intraocular inflammation and vision compromise [23].

### 2.7. Behaviour

Behaviour of birds was monitored in males only, using infra-red video recording equipment to allow 24 h observations. 

**Figure 6:**
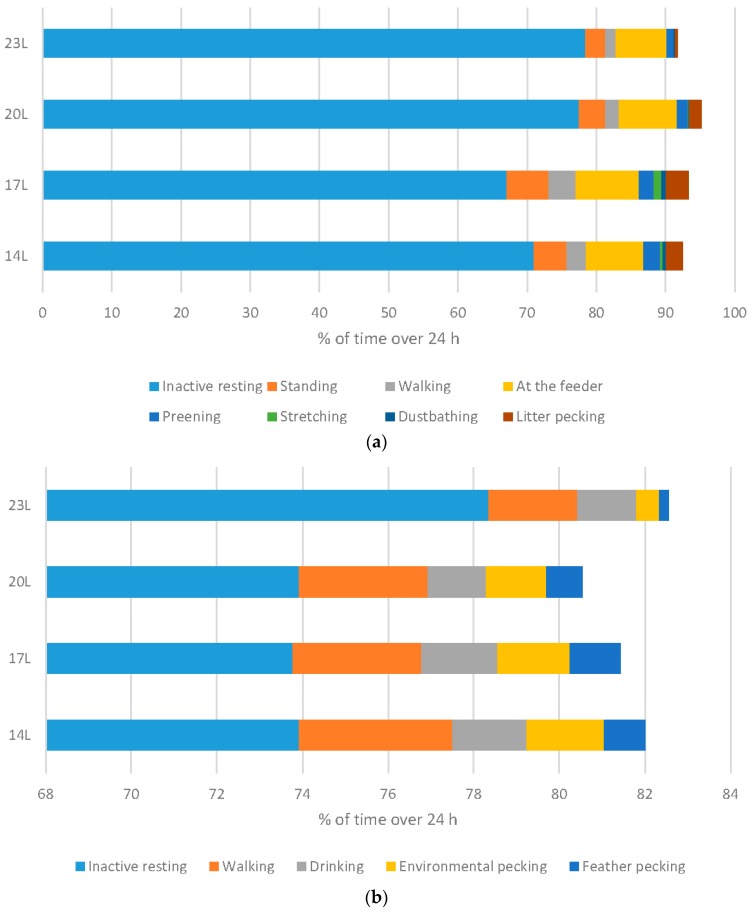

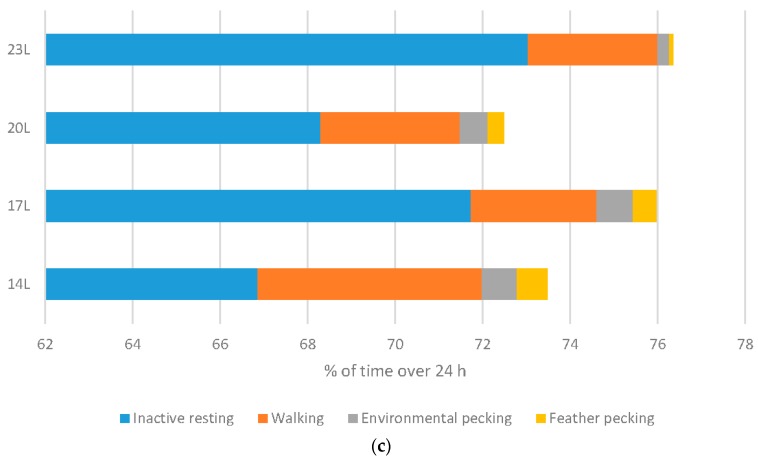
The impact of daylength on behaviour (percent of time). (**a**) Behavioural activities of broilers—percent of time differing significantly by photoperiod over 24 h period at 27/28 d of age; (**b**) Behavioural activities of turkeys—percent of time differing significantly by photoperiod over 24 h period at 11 weeks of age; (**c**) Behavioural activities of turkeys—percent of time differing significantly by photoperiod over 24 h period at 17 weeks of age.

Broilers: Increasing the daylength for broilers resulted in a significant change to the ethogram of the birds. Measuring behavioural output over a 24 h period clearly indicated that birds are much less responsive, and as a result spend more time inactive resting, and less time in exercise behaviours such as walking and standing (both linear responses), less time at the feeder (quadratic response), and less time in both exploratory and comfort behaviours (preening, stretching, dustbathing (linear responses) and foraging (quadratic response) (Figure 6a).

Turkeys: Daylength had a similar influence on turkey behaviour as it did on broiler behaviour (Figure 6b). A linear relationship between daylength and the percentage of time turkeys spent inactively resting when monitored at 11 weeks of age. Conversely, 23 L turkeys also spent less time walking. Exploratory behaviours were reduced under 23 L at this age, with birds reared under long daylengths spending less time environmental or feather pecking. Interestingly, no difference in the percent time feeding occurs under different daylengths. At older ages, statistical differences were only noted in time spent walking (linear with less activity under long daylengths), and environmental and feather pecking (linear with less activity under long daylengths) (Figure 6c).

The use of long daylength for broilers and turkeys reduced activity in general, which may be a reason that some producers choose to use this photoperiod length. Long daylength changes the behaviour output, with less comfort, exploratory, and exercise behaviours expressed. Together, these changes suggest a reduction in welfare with regards to behaviour, and health, as exercise itself can improve metabolic and skeletal functioning. 

### 2.8. Circadian Rhythms and Patterns

Broilers: Broiler flocks exposed to 23 L did not demonstrate circadian patterns in a number of response variables [20], including melatonin production and behaviours, including resting, feeding, dustbathing, walking and others. While weak rhythms were noted in broilers reared on 20 L, strong circadian rhythms were found in flocks exposed to either 7 or 10 h of darkness per day. Furthermore, the range between the minimum and maximum levels was the least in flocks reared under 23 L (Table 1). 

Turkeys: Melatonin production was not measured in the turkey experiment. Examination of the behavioural rhythms showed similar responses of turkeys to the graded daylengths as was seen in broilers in feeding and inactive resting, with no rhythms occurring in turkeys reared on 23 L and only weak rhythms on those reared under 20 L.

## 3. Conclusions

The use of light in poultry production systems can have powerful impacts on many productivity and welfare traits, and the comparison in this paper aids in the understanding that the responses are not always identical between species. Other light components may be equally important. For example, light intensity can impact broiler behaviour [48,49,50], with low light intensities generally resulting in less active birds similar to the responses noted here on long daylengths. Interestingly, low light intensity also results in an increase in eye weight of broilers similarly to the impact of long daylengths [50,51], possibly signaling that distinction between day and night is also important in the regulation of circadian rhythms.

The data from these two projects clearly indicate that using near-constant light (23 L:1 D) has significant negative impacts on the welfare of both broilers and turkeys. Interestingly, production (body weight in both species and feed efficiency in broilers) does not benefit from long daylengths. A multitude of reasons for the negative impact on bird welfare have been discussed in this paper, including shifting of the time of weight deposition, and increased exercise levels. 

Another theory is that broilers and turkeys exposed to long daylengths may be suffering from sleep deprivation. When the broiler data included in this review were analyzed for flock behavioural rhythms [20], it was evident that no rhythms existed for behaviours such as feeding, exercise or resting when long daylengths were used. Flock melatonin circadian rhythms also did not exist [20]. These factors suggest that sleep fragmentation may be occurring, as flock circadian rhythms in resting were not present for either species under 23 L. Therefore if birds are attempting to sleep during the long days, constant disruptions from other birds result in repeated awakening, with the impact mimicking total sleep deprivation [13,52,53]. 

The remaining data support this hypothesis for both species. Sleep deprivation impacts brain function, resulting in reduced alertness [10]. Both species showed a decreased level of activity, and a reduction in the expression of other behaviours when reared on long daylengths as compared to those including a dark period. It has been shown in humans [54] and rats [55] to impact digestive efficiency. Sleep and the associated melatonin rhythms have been linked to reduced health and a reduction in tissue regeneration [11], and both species clearly had higher mortality and morbidity levels with longer daylengths. The lack of melatonin rhythms could disrupt normal eye growth, and that again was noted in both species. In this case, the impact on turkeys was likely more severe because of the length of the period the birds are exposed to the lighting program, although the authors cannot rule out other factors such as sensitivity of the turkey eye.

In turkeys, a conflicting point with regards to using short daylengths is the increase in pecking that is noted. While an increased incidence of pecking, particularly environmental pecking, may signify improved positive welfare, and may be related to reduced eye pathology or overall health improvements, it can also reduce well-being, as aggressive pecking continues to be a concern in turkey production.

It should be noted that in the studies reported here, only one genotype of turkeys, and broilers from one breeding company, were tested. Unpublished lighting data from the same lab testing similar photoperiods over multiple lines of broilers, found that in general, strains respond similarly to exposure to varying daylengths. Therefore, the authors believe that use of long daylengths significantly reduces the well-being of broilers and turkeys, and for the latter species, the welfare challenges may be magnified because of the length of time spent in the environment (*i.e.*, ocular health). The amount of darkness used to reach maximum well-being may differ slightly between bird types. For example, mortality and morbidity is the lowest on broilers given 7 to 10 hours of darkness, but turkey mortality is similar with a darkness exposure of 4–10 hours per day. Therefore, it may be beneficial to base the darkness requirement on the particular species, and within the turkey population, with consideration given for the gender.

## Figures and Tables

**Table 1:** The impact of daylength on broiler minimum and maximum daily melatonin levels (pg/mL) and the range between minimum and maximum values [20].

Melatonin (pg/mL)	Daylength (h)				SEM
	14	17	20	23	
Minimum	29.5 ^C^	39.8 ^B,C^	51.2 ^A,B^	53.2 ^A^	2.80
Maximum	119.7 ^B^	112.3 ^B^	149.5 ^A^	98.0 ^B^	5.69
Range	90.2 ^A^	72.5 ^A,B^	98.3 ^A^	44.9 ^B^	6.35

^A,B,C^ Means within a row lacking a common superscript vary significantly (*p* ≤ 0.05); SEM: standard error of the mean.
